# In Vitro Innervation as an Experimental Model to Study the Expression and Functions of Acetylcholinesterase and Agrin in Human Skeletal Muscle

**DOI:** 10.3390/molecules22091418

**Published:** 2017-08-27

**Authors:** Katarina Mis, Zoran Grubic, Paola Lorenzon, Marina Sciancalepore, Tomaz Mars, Sergej Pirkmajer

**Affiliations:** 1 Institute of Pathophysiology, Faculty of Medicine, University of Ljubljana, Zaloška 4, SI-1000 Ljubljana, Slovenia; katarina.mis@mf.uni-lj.si (K.M.); tomaz.mars@mf.uni-lj.si (T.M.); 2 Department of Life Sciences, University of Trieste, via A. Fleming 22, I-34127 Trieste, Italy; plorenzon@units.it (P.L.); msciancalepore@units.it (M.S.)

**Keywords:** acetylcholinesterase, in vitro innervation, human muscle, neuromuscular junction, co-cultures, agrin, apoptosis

## Abstract

Acetylcholinesterase (AChE) and agrin, a heparan-sulfate proteoglycan, reside in the basal lamina of the neuromuscular junction (NMJ) and play key roles in cholinergic transmission and synaptogenesis. Unlike most NMJ components, AChE and agrin are expressed in skeletal muscle and α-motor neurons. AChE and agrin are also expressed in various other types of cells, where they have important alternative functions that are not related to their classical roles in NMJ. In this review, we first focus on co-cultures of embryonic rat spinal cord explants with human skeletal muscle cells as an experimental model to study functional innervation in vitro. We describe how this heterologous rat-human model, which enables experimentation on highly developed contracting human myotubes, offers unique opportunities for AChE and agrin research. We then highlight innovative approaches that were used to address salient questions regarding expression and alternative functions of AChE and agrin in developing human skeletal muscle. Results obtained in co-cultures are compared with those obtained in other models in the context of general advances in the field of AChE and agrin neurobiology.

## 1. Introduction 

Acetylcholineesterase (AChE) and agrin, a heparan sulfate proteoglycan, are important for termination of neuromuscular transmission and maintenance of the neuromuscular junction (NMJ), respectively [1,2,3,4,5,6]. In addition to these canonical roles in NMJ, AChE and agrin have alternative functions that are not related to NMJ and cholinergic transmission [7,8,9,10,11]. Canonical and alternative roles of AChE and agrin have been investigated using many different in vitro and in vivo experimental approaches. Comprehensive overview of all these approaches is beyond the scope of this review, and here we focus solely on the challenges of investigating molecular mechanisms underlying expression and function of AChE and agrin in human skeletal muscle. Indeed, the major challenge for this type of studies is a lack of suitable human models. 

First of all, aside from investigating effects of pharmacological agents that affect neuromuscular transmission [12], in vivo studies on human NMJ, especially its synaptogenesis, are almost impossible to conduct. Similarly, ex vivo models, such as preparations of the rat phrenic nerve and the diaphragm muscle, which can be used to assess different aspects of NMJ function [13,14,15], or isolated strips of human skeletal muscles, which yielded important insights into muscle metabolism [16,17,18,19,20], are not easily adaptable for experimental manipulation of human NMJ. Furthermore, under most circumstances, a scarcity of human embryonic cells and tissues and ethical concerns regarding their use also prevents approaches that enable the investigation of NMJ synaptogenesis and function using animal embryonic material. In addition, unlike various skeletal muscle cells of animal origin, aneurally cultured human cells, the most widely used in vitro model to study human skeletal muscle [21], under standard conditions usually do not spontaneously contract or form differentiated postsynaptic components of NMJ [22,23]. 

An elegant solution to these challenges is provided by co-culture with embryonic rat spinal cord, which enables establishment of functional innervation of human skeletal muscle cells in vitro [23,24,25]. The model of in vitro innervation enables examination of NMJ synaptogenesis as well as different aspects of AChE and agrin function in contracting human skeletal muscle cells that possess functional NMJs. In this review, we provide description of biological characteristics and methodological aspects of this model and discuss them in the context of AChE and agrin neurobiology. 

## 2. The Experimental Model of the in Vitro Innervated Human Skeletal Muscle Cells

### 2.1. The Development of Skeletal Muscle is More Nerve-Dependent in Humans than in Animal Species Used in Skeletal Muscle Research

Most of the research in the field of medicine has been performed on various animal models with the expectation that the obtained results could be extrapolated, at least to some extent, to humans. Among several limitations of these extrapolations are the biological differences between human skeletal muscle and the skeletal muscles of frogs, chicks and rodents, which are the most frequently used animal models in skeletal muscle research. These differences are evident already at the level of primary skeletal muscle cell cultures. Neuromuscular transmission represents the only physiological path to trigger contractions of skeletal muscle fibers under in vivo conditions. At the beginning we will therefore describe the development of postsynaptic components of NMJ and contractile properties in aneurally cultured human skeletal muscle cells in comparison with animal cells. 

#### 2.1.1. Postsynaptic NMJ Components in Aneurally Cultured Human and Animal Skeletal Muscle Cells 

Under in vivo conditions AChE is bound to the basal lamina of NMJ and nicotinic acetylcholine (ACh) receptors (nAChRs) are concentrated in the postsynaptic membrane of NMJ [2,3,26]. Aneurally cultured human skeletal muscle cells express AChE [27] and nAChRs diffusely [23,24,25]. Furthermore, in contrast to cultured embryonic animal myotubes [28], human myotubes [24] lack basal lamina; i.e., the structure, which is not only a major component of the NMJ synaptic cleft, but also provides binding sites for AChE and agrin [2,29]. Skeletal muscle cells of animal origin display a more advanced level of postsynaptic organization than human cells. For instance, despite the absence of neurons, clusters with high concentration of nAChRs are often present in aneurally cultured embryonic chick [30,31], rat [32] and mouse [33] myotubes. 

Sanes’s group showed that postsynaptic differentiation of NMJ in C2C12 myotubes, a widely used mouse skeletal muscle cell line, is nerve-independent [34]. In order to get a more detailed insight into species-specific differences in NMJ synaptogenesis, we examined whether postsynaptic differentiation of NMJ in cultured human myotubes can also be induced in the absence of motor neurons. We compared effects of laminin and gelatin coating on formation of differentiated pretzel-like postsynaptic clusters of nAChRs in aneural C2C12 and human myotubes [35]. Consistent with the results from Sanes’s group [34], we found that laminin coating significantly increased the number of highly differentiated nAChR clusters in aneurally cultured C2C12 myotubes. However, we also found that the presence of laminin was not necessary for formation of differentiated nAChR clusters, since they formed also in C2C12 myotubes grown on gelatin coating. Conversely, in cultured human myotubes laminin or gelatin coating was unable to promote formation of nAChR clusters. Indeed, human myotubes displayed well-developed nAChR clusters only once innervated by neurites that extended from the rat embryonic spinal cord (see description of the co-culture model below). Furthermore, nAChR clusters developed exclusively in the areas where neurites made contact with myotubes [35], underscoring the role of innervation in postsynaptic differentiation of NMJ in human skeletal muscle cells.

Our experiments clearly demonstrate that innervation is crucial for nAChR clustering in human myotubes. Formation of nAChR clusters as well as other processes involved in postsynaptic differentiation of NMJ require activation of the muscle-specific kinase (MuSK) [36,37,38]. Notably, MuSK mRNA expression levels are ~1000-fold higher in C2C12 myotubes than in human myotubes [35]. Given that overexpression of MuSK can lead to its spontaneous activation [39], these data suggest a mechanism whereby spontaneous MuSK activity in C2C12 myotubes is sufficiently high to induce nAChR clustering in the absence of additional exogenous stimuli. In aneural C2C12 myotubes, laminin therefore acts primarily as facilitator or enhancer of this intrinsically active differentiation process. Indeed, laminin does not stimulate nAChR clusters in muscles cells that lack MuSK or express mutant MuSK without its extracellular domain [34,40]. Similarly, nAChR clusters do not form despite the presence of laminin in C2C12 cells lacking rapsyn [34], an intracellular membrane protein that mediates effects of MuSK on AChR clustering [1,41]. We may therefore surmise that expression and activity of MuSK in aneurally cultured human myotubes are too low to stimulate accumulation of nAChR and other aspects of postsynaptic NMJ differentiation even in the presence of laminin. 

When considered together, these data indicate that MuSK can be sufficiently activated in human myotubes only once they are innervated by motor neurons and basal lamina is formed in the synaptic cleft [42]. Basal lamina therefore likely enables neural agrin, secreted by motor neurons, to occupy its optimal position in the synaptic cleft from where it can effectively bind to its receptor, the low-density lipoprotein receptor-related protein 4 (Lrp4) [43,44], thus enhancing Lrp4-MuSK interaction and activation of MuSK [1] in cultured human myotubes. 

#### 2.1.2. Contractile Properties of Aneurally Cultured Human and Animal Skeletal Muscle Cells

Under conventional culture conditions, primary human myotubes usually contract only if they are innervated [24,27,35,45,46] or electrically stimulated [47,48,49]. In some cell culture systems, primary human myotubes contract if they are treated with ACh [50]. Aneurally cultured human myotubes mostly display immature contractile apparatus, including poorly developed sarcomeres, cross-striations and T-tubular system [24]. Conversely, cultured primary chick [51,52,53,54], quail [55,56], rat [57,58,59,60,61,62] and mouse [34,63,64] skeletal muscle cells, which are often obtained from embryos or neonatal animals, display more advanced level of differentiation and contract spontaneously in the absence of innervation or electrical stimulation. Indeed, to study quiescent primary rat or mouse skeletal muscle cells tetrodotoxin (TTX), a blocker of voltage-gated Na^+^ channels, is used to suppress spontaneous contractions [34,60,61,62,65,66,67]. Spontaneous discharge of action potentials provides one explanation for autonomous contractile activity of aneurally cultured skeletal muscle cells [51]. Interestingly, cultured mouse myotubes may contract autonomously (i.e., independent of innervation) even in the presence of motor neurons [68]. 

Spontaneous contractile activity, as well as the presence of nAChR clusters in animal myotubes, has been successfully exploited to examine effects of electromechanical activity on expression and distribution of AChE and nAChR. Indeed, by using animal embryonic cells it was possible to study effects of TTX-induced quiescence as well as spontaneous and/or electrically-stimulated muscle contractile activity on the expression of AChE [60,61,65] and nAChR [51,57,62,69,70]. For instance, expression of the 16S (A12) AChE, the major NMJ AChE species [5,71], is markedly increased by spontaneous contractile activity of rat [60,61], mouse [67] and quail [56] myotubes. On the other hand, in cultured human skeletal muscle cells expression of the 16S (A12) AChE, was found only in innervated co-cultures [22]. In contrast to AChE, electromechanical activity of rat myotubes reduces expression of several nAChR subunits [69,70]. Importantly, stimulation of nAChR with carbachol was shown to lead to the loss of nAChR clusters in cultured rat myotubes [72]. This effect was later used to demonstrate that the neural isoform of agrin prevents activity-induced loss of nAChR clusters [73].

Since electrical stimulation was successfully employed to produce contracting human myotubes [47,48,49,74], electrical activity apparently suffices for acquisition of functional contractile apparatus. However, development of functional contractile apparatus and postsynaptic differentiation of NMJ are not necessarily directly coupled. For instance, primary mouse myotubes display highly developed nAChR clusters despite treatment with TTX to suppress spontaneous contractions [34]. C2C12 myotubes, which also display highly developed nAChR clusters [34], may contract spontaneously [75,76], but under standard conditions often fail to do so [77] unless they are exposed to electrical [78] or pharmacological stimulation of nAChR [79,80]. Notably, MuSK and rapsyn are required for formation of AChR clusters in electrically stimulated embryonic *Xenopus* myotubes in vitro [81]. Whether electrical stimulation-induced assembly of functional contractile apparatus in human myotubes is associated with MuSK/rapsyn-dependent postsynaptic differentiation of NMJ, such as clustering of nAChRs, remains to be characterized. 

#### 2.1.3. Evolutionary Basis for Differences in Developmental Programme of Skeletal Muscle Cells?

Failure of aneural human myotubes to develop functional, spontaneously active, contractile apparatus and differentiated, pretzel-like, nAChR clusters demonstrates that developmental programming of human skeletal muscle cells is highly dependent on innervation by motor neurons. This innervation-dependent program is contrasted by intrinsic capability of embryonic animal skeletal muscle cells to achieve high degree of differentiation without innervation. One possible reason for these differences is inability of standard cell culture plates and media to support further development of human skeletal muscle cells due to a lack of specific extracellular matrix components or growth factors. However, while agrin or the conditioned medium collected from co-cultures of contracting human myotubes and embryonic rat spinal cord promote nAChR clustering in aneural human myotubes [82,83], such treatment does not seem to be capable of fully supporting their further differentiation into spontaneously contracting myofibers. This implies that secreted factors are not sufficient and that human skeletal muscle cells require physical contact with motor neurons, formation of functional NMJ and/or subsequent electromechanical activity to continue with their intrinsic developmental program. Indeed, even in co-cultures, where high concentrations of nerve-derived factors can be expected, myotubes that are not innervated by motor neurons do not further differentiate and ultimately degenerate [24]. 

Regardless of the underlying mechanism NMJ development of cultured human skeletal muscle cells is clearly more nerve-dependent, than development in avian and rodent skeletal muscle cells, which is much more autonomous. These species-specific characteristics of skeletal muscle development may reflect differences in organization of motor systems. Fractionation of movement, a number of independent and individual movements that can be performed, is the most developed in humans. The capability of performing refined movements in humans is paralleled by prominently developed direct, i.e., monosynaptic, connections between cortical neurons and α-motor neurons [84,85]. With some rare exceptions [86] in non-primate species, cortical neurons regulate α-motor neurons via indirect, polysynaptic, connections [85]. Monosynaptic connections between cortical neurons and α-motor neurons enable more focalized regulation of the motor unit activity, which in turn results in more refined regulation of movement. Innervation-dependent development of postsynaptic NMJ differentiation in human skeletal muscle cells therefore seems to be consistent with imposition of more direct and focalized neural control over skeletal muscle activity. According to this idea, NMJs develop exclusively in human skeletal muscle cells that are contacted by nerve endings of motor neurons, thus leading to their further development into contractile myotubes (myofibers), while uninnervated skeletal muscle cells are rapidly eliminated. Notably, such sequence of events, including the tendency to develop monosynaptic innervation (i.e., one NMJ per myotube), occurs in co-culture of human skeletal muscle cells and embryonic rat spinal cord [23,24,25,35]. 

### 2.2. Experimental Model of the in Vitro Innervated Human Skeletal Muscle Cells: Preparation and Description

Functioning of the nervous system is based on the synaptic communications among its cellular constituents. A great deal of scientific effort in neuroscience has therefore been focused on the mechanisms underlying this communication. NMJ is relatively easy accessible to observation and experimental manipulation. Since basic mechanisms of neurotransmission and synaptogenesis are relatively similar between different types of synapses, data obtained in various NMJ experimental models can be extrapolated, with some caveats and limitations, to other synapses (reviewed in [87,88]). NMJ has therefore been used as a model synapse, which provided the basis for understanding synaptogenesis and synaptic communication in general.

#### 2.2.1. Co-Culture Models to Study NMJ

The mechanisms of NMJ synaptogenesis and synaptic communication have been approached in various in vitro models, which enable simple identification and monitoring of different stages of NMJ synaptogenesis. In vitro models of NMJ are co-cultures that require the nervous and the skeletal muscle component. The possibilities for the nervous component include explants of embryonic spinal cord, isolated ganglia, dissociated ganglionic cholinergic neurons, or dissociated motor neurons [24,89,90,91,92,93]. Alternatively, neuroblastoma-glioma hybrid cells [33,94] or pheochromocytoma cells can be used to study cholinergic neurotransmission [95]. The possibilities for the skeletal muscle component are also varied and include myofibers [96], primary skeletal muscle cells derived from muscle satellite cells [23,24,25,97], embryonic skeletal muscle cells [98,99,100] as well as immortalized skeletal muscle cell lines [89,101,102]. The source of neurons and skeletal muscle cells is important since it affects different aspects of NMJ synaptogenesis, such as the timing of de novo NMJ formation as well as its subsequent stability and extent of differentiation [46]. For instance, if explanted human muscle fibers are used basal lamina is not formed de novo [96]. Conversely, a new basal lamina is formed upon innervation if primary human skeletal muscle cells are used to prepare the co-culture [24]. 

In homologous models the nervous and the skeletal muscle component are from the same species, while heterologous models contain components from different species. Many different homologous and heterologous co-culture models have been described to study innervation of animal skeletal muscle cells. However, as regards in vitro innervation of human skeletal muscle cells, heterologous co-culture with embryonic rat spinal cord has been the most widely used approach [9,23,24,25,27,35,42,46,82,103,104,105,106,107,108,109]. Alternative approaches to innervate human skeletal muscle cells include co-culture with embryonic mouse spinal cord [110], ventral part of embryonic rat spinal cord [111] or dissociated embryonic rat spinal cords [22]. In addition to these well-established traditional models, homologous human models were also established. For instance, homologous co-culture can be established by using spinal stem cells derived from human fetus and primary skeletal muscle cells [112]. Another possibility is to use induced pluripotent stem cell (iPSC) technology, which enables generation of human motor neurons [50,113] as well as skeletal muscle cells [113,114,115]. The iPSC-derived human motor neurons can form NMJs with the iPSC-derived human myotubes [113] as well as with primary human [50] or C2C12 [101] myotubes. In the continuation we focus on the rat-human co-culture model, while providing relevant comparisons with other human or animal models.

#### 2.2.2. Co-cultures of Primary Human Skeletal Muscle Cells and Rat Embryonic Spinal Cord: Basic Characteristics

**Figure 1:**
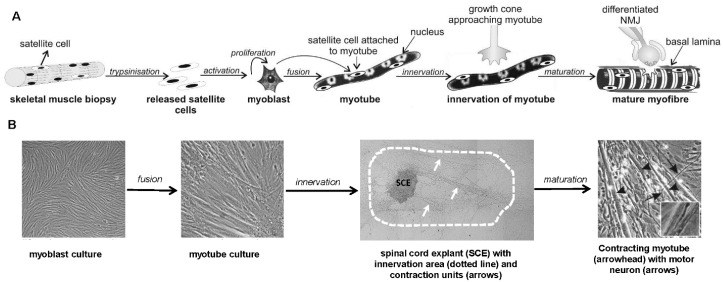
Schematic presentation of the stages of skeletal muscle development and co-culture preparation (**A**) and micrographic illustration of co-culture stages (**B**). **A**. Stages of skeletal muscle development as followed in co-culture system. Myoblasts are derived from satellite cells released from muscle biopsy; these proliferate further and subsequently fuse to myotubes. Myotubes are innervated by neurites growing from the embryonic rat spinal cord explants and mature into innervated contracting myotubes, thus giving rise to myofibers. **B**. Micrographic illustrations of co-culture stages: mononucleated myoblasts, multinucleated myotubes, myotubes co-cultured with embryonic rat spinal cord explant with innervation area; mature innervated and contracting myotube with cross striations seen at higher magnification (insert). Adopted from [42,46,132]. For definition of the contracting unit see Table 1.

Co-culture of embryonic rat spinal cord explants with primary human skeletal muscle cells was introduced to NMJ research by Askanas and her group [23,24,25]. In this model, rat embryos are dissected to obtain spinal cord explants, which are needed as a source of motor neurons, while human skeletal muscle cells are obtained from biopsies or discarded surgical material. The preparation of co-cultures and time course of its development are schematically depicted in Figure 1A (technical aspects are reviewed in [109]). The typical co-culture with spinal cord explant and contraction units is shown in Figure 1B. The heterologous, rat-human co-culture model, which uses primary human skeletal muscle cells, has four major characteristics which make it particularly suitable for in vitro investigation of NMJ formation and neuromuscular transmission.

The first characteristic is that human skeletal muscle cells do not, under most circumstances, contract unless they are innervated by motor neurons [21,22,23,24,27,45]. Formation of NMJs leads to functional neuromuscular transmission, which drives contractions of muscle cells. Indeed, electrophysiological studies demonstrated miniature as well as stimulus-evoked endplate potentials [116,117]. Furthermore, contractions of human myotubes cease in the presence of NMJ blockers that prevent ACh action at the nAChR, such as nAChR antagonists tubocurarine, α-bungarotoxin [24] or rocuronium [118]. Importantly, block induced by rocuronium can be completely reversed by the addition of sugammadex [118], which binds to rocuronium and thereby prevents its effects on nAChR. These experiments underscore that contractions of innervated human myotubes are not autonomous (i.e., nerve-independent) but are triggered by release of ACh from motor neuron at the NMJ. Thus, contractions observed in rat-human co-cultures are triggered by neuromuscular transmission, which enables quantitative analyses of functional innervation [46] (see Figure 1 and Table 1). 

Although embryonic rat [57,58,59,60,61,62], mouse [34,63,64] and chick [51,52,53,54] myotubes may develop vigorous contractions in the absence of neurons, very useful co-culture models using animal skeletal muscle cells have also been developed. For instance, in a homologous mouse-mouse model, which uses explants of embryonic mouse spinal cord and skeletal muscle, contractions of skeletal muscle cells are completely suppressed by succinylcholine (aka suxamethonium) [99,100], a depolarizing nAChR agonist and an NMJ blocker. Contractions in this model are also suppressed by nAChR antagonist rocuronium [119], as well as by AChE inhibitors, such as soman and VX [100,120], and by botulinum toxin [98], which blocks release of ACh from presynaptic terminals. Furthermore, in a homologous rat-rat model, in which both spinal cord and skeletal muscle cells are obtained from rat embryos, the pattern of contractions is modulated by application of strychnine and bicuculline, which antagonize effects of inhibitory neurotransmitters glycine and GABA, respectively, as well as by electrical stimulation of spinal cord explants [68], which underscores the role of motor neurons in driving the muscle activity. The great majority of muscle contractions in homologous models using embryonic animal tissues therefore seem to be driven by motor neuron activity despite the ability of skeletal muscle cells to contract autonomously. However, in the homologous rat-rat model, muscle contractions were suppressed, but not completely abolished, by the application of nAChR antagonist tubocurarine [68,121]. Some contractions are also not modulated by strychnine, bicuculline, CNXQ (an antagonist of excitatory neurotransmitter glutamate) or electrical stimulation of the spinal cord explant, thus further indicating that not all contractions are the result of neuronal activity [68]. Thus, a minor fraction of contractions is independent of neuromuscular transmission [68], which is consistent with spontaneous electromechanical activity of aneurally cultured embryonic muscle cells.

The second important characteristic is that heterologous models can be used to assess the origin of synaptic proteins simply by using wild type cells. Indeed, in the rat-human model all proteins produced in the nervous tissue are of rat origin, while all proteins produced in skeletal muscle cells are of human origin. By using species-specific antibodies, such chimeric expression pattern can be exploited to investigate whether various components of the synaptic cleft, such as AChE, are nerve- and/or muscle-derived [122]. Similarly, species-specific antibodies were used in co-culture of chick skeletal muscle cells and dissociated rat neurons to determine that, in addition to motor neurons, skeletal muscle cells also secrete agrin into the synaptic cleft of NMJ [123].

Third, the heterologous rat-human model uses skeletal muscle cells obtained from muscle biopsies. On the one hand, the use of primary human skeletal muscle cells avoids some of the challenges associated with extrapolation of data obtained in animal skeletal muscle cells or the iPSC-derived human skeletal muscle cells. Furthermore, by obtaining myogenic cells from diagnostic muscle biopsies, genetic neuromuscular disorders can be directly studied in cell culture. Such approach was used to investigate biological characteristics of innervated skeletal muscle cells in various neuromuscular diseases, such as McArdle’s disease (deficiency of the muscle glycogen phosphorylase) [124], Duchenne muscular dystrophy [125], myotonic dystrophy [126], spinal muscular atrophy [127,128] and X-linked myotubular myopathy [129].

Finally, the rat-human model enables long-term studies on innervated human myotubes. Indeed, in addition to being markedly more developed, innervated myotubes can be maintained in culture for 6 months, while aneural myotubes do not survive beyond 6–8 weeks [24,25]. Long-term studies are important because some innervation-induced alterations, such as expression of metabolic enzymes, may gradually evolve over many weeks [124,130,131]. Longevity of co-cultures might be particularly useful for examination of molecular mechanisms underlying pathogenesis of neuromuscular disorders.

#### 2.2.3. Developmental Characteristics of the in Vitro Innervated Human Skeletal Muscle Cells 

Functional maturation of innervated human skeletal muscle cells is evident at several levels. First of all, innervation leads to development of highly differentiated and functional NMJs [24]. Establishment of NMJs is paralleled by alterations in AChE and nAChR expression and distribution. While aneural myotubes express AChE mRNA in most nuclei, innervated myotubes express it mostly in nuclei close to the NMJ [27]. In innervated myotubes, AChE and nAChR are concentrated at NMJ, while their abundance is low in extrajunctional sites [23,25,27]. In vitro innervation therefore induces functional diversification of myonuclei and formation of junctional AChE and nAChR patches. These events are paralleled by de novo development of basal lamina, to which agrin and AChE bind in myofibers in vivo [2,3,29]. Furthermore, innervated myotubes tend to make transition from polysynaptic towards monosynaptic innervation [25]. Thus, while co-culture clearly cannot fully mimic in vivo conditions, innervated myotubes resemble mature myofibers, which display high concentration of AChE and nAChR at a single NMJ containing basal lamina [2]. 

Maturation of innervated human skeletal muscle cells is evident also at the level of cytoskeleton. Sarcomeres and the T-tubular system in innervated myotubes display a more advanced stage of organization than in aneural myotubes [24]. Under in vivo conditions different types of muscle fibers are characterized by expression of specific myosin heavy chains (MyHC). Type I (slow) fibers, which are highly oxidative and resistant to fatigue, express predominantly MyHC-β/slow (*MYH7* gene) and type IIA (fast) fibers, which are oxidative-glycolytic and fatigue resistant, express predominantly MyHC 2A (*MYH2* gene) [133,134]. Type IIX (fast) fibers, which are glycolytic and fast fatigable, express MyHC 2X (*MYH1* gene) [133,134]. Aneurally cultured human myotubes express *MYH1*, *MYH2* and *MYH7* [47]. Several studies investigated sarcomere ultrastructure [24] and MyHC expression in innervated human skeletal muscle cells [135,136]. Two major conclusions can be drawn from these studies. First, while different MyHCs are expressed in innervated as well as aneural human myotubes, only innervated myotubes consistently display well-developed cross-striations [24]. Second, innervation of cultured skeletal muscle cells induces assembly of myosin into myofilaments, thus leading to formation of functional contractile apparatus. While these conclusions are consistent across different studies, investigation of MyHC expression pattern produce divergent results, which can partly be explained by usage of different anti-MyHC antibodies and different models of in vitro innervation [111,135,136]. However, as assessed by PCR, immunoblotting and immunofluorescence innervated human myotubes express *MYH1*, *MYH2* and *MYH7* (unpublished observations T.M., K.M., S.P. and Nataša Nikolic). 

In addition to maturation of myofilaments, innervated skeletal muscle cells display a continuous subsarcolemmal distribution of dystrophin. Conversely, distribution of dystrophin in aneural cells is patchy [125]. Aside from morphological and functional maturation of NMJ and cytoskeleton, in vitro innervated skeletal muscle cells display biochemical characteristics resembling those in mature myofibers. For instance, the innervated cells have higher expression of the muscle-specific isoforms of creatine kinase (CK-MM), glycogen phosphorylase, lactate dehydrogenase and phosphoglycerate mutase than aneural cells [104,106,124,130]. 

Innervation also promotes maturation of electrophysiological properties of cultured skeletal muscle cells [117,137]. First, in innervated contracting myotubes resting membrane potential is lower, and therefore closer to that of mature myofibers, than in aneural myotubes [117]. Second, treatment with caffeine or high concentrations of KCl, which trigger transient increases in intracellular Ca^2+^ concentration, are less dependent on extracellular Ca^2+^ in innervated than in aneural myotubes [137]. Excitation-contraction coupling in vertebrate skeletal muscle is typically independent of extracellular Ca^2+^ [138,139]. Innervated myotubes therefore display more mature excitation-contraction coupling mechanism than aneural myotubes. Finally, innervated myotubes contract in response to caffeine, which stimulates ryanodine receptors and thereby triggers release of Ca^2+^ from sarcoplasmic reticulum, as well as KCl-induced depolarization, while aneurally cultured myotubes fail to do so [137], underscoring that functional and morphological maturation of contractile apparatus develop in parallel. 

#### 2.2.4. Challenges and Limitations of Using the Coventional Rat-Human Co-Culture Model

Challenges and limitations of the co-culture model can be divided into two broad categories: biological and technical. From the biological perspective, immaturity of skeletal muscle cells and NMJ needs to be taken into consideration. Innervated human skeletal muscle cells are markedly more developed than aneural cells; however, they cannot be considered fully mature. For instance, concentration of non-muscle isoforms of creatine kinase, such as CK-BB, is relatively higher in co-cultured cells than in samples from muscle biopsies [130]. Also, while innervation promotes expression of the A12 AChE [140], it represents a relatively low fraction of total AChE activity even after 8 weeks of co-culture [140]. Indeed, average activity of the A12 AChE in the rat-human co-cultures represents approximately 9% of the total AChE activity [140], consistent with observations in the homologous chick co-culture model [141]. Furthermore, even after weeks of innervation in vitro NMJ and the T-tubular system do not attain the organizational level of mature myofiber in vivo [24]. Innervated myotubes are also thinner than normal myofibers [25]. Their diameter is usually not much bigger than diameter of myonuclei, which therefore occupy almost the entire thickness of the innervated myotubes. Nevertheless, some myonuclei in innervated myotubes assume the subsarcolemmal position [129].

One of the challenges in traditional cell culture is also absence of normal tissue architecture. Haphazard growth of skeletal muscle cells under traditional cell culture conditions may adversely affect their potential for fusion and NMJ formation. Possible solutions include the use of various patterned substrates, which direct the growth of myotubes and/or motor neurons, and three dimensional culture systems [101,142,143,144,145]. However, even aneurally cultured myotubes, which are frequently used to investigate various aspects of skeletal muscle physiology and pharmacology, reflect properties of skeletal muscle [21] despite displaying lower degree of maturity than innervated myotubes. Thus, innervated human myotubes can be regarded as a valid in vitro model for investigation of human skeletal muscle function.

From the technical perspective, heterogeneity of co-cultures needs to be considered. Indeed, co-cultures encompass innervated myotubes, non-innervated myotubes, various non-myogenic cells originating from skeletal muscles as well as spinal cord explants. On the one hand, the presence of different types of cells is beneficial for the function of cultured skeletal muscle cells and/or motor neurons [25,45,146]. Also, despite heterogeneity the co-culture model is very suitable to conduct morphological, such as electron microscopy [24], or electrophysiological studies [117,137] since analyses can be selectively conducted on innervated myotubes. Conversely, heterogeneity provides a challenge for analyses which require to work with homogenates. For instance, results of metabolic assays, such as glucose uptake, will reflect to a certain extent the metabolism of skeletal muscle cells as well as the metabolism of non-muscle cells. Similarly, western blot analyses are a challenge whenever species-specific antibodies are not available and target proteins are expressed in skeletal muscle as well as nervous and other non-myogenic cells. A similar problem arises with PCR analyses if 18S rRNA is used as an endogenous control, since gene expression assays then detect 18S rRNA from human as well as rat origin. One approach to circumvent these challenges is to use single-cell PCR [147,148]. Another possibility is to perform in situ hybridization [27]. In addition, compartmentalized cell culture models, which partially separate the nervous and the muscle component of co-culture, such as microfluidic chamber, allow more selective experimental manipulations and analyses [63,101] than conventional co-culture system, in which spinal cord explants are plated directly on monolayer of skeletal muscle cells.

Finally, while rat embryonic spinal cord is easier to work with than mouse embryonic spinal cord due to size differences between rat and mouse embryos, transgenic mouse models are more widely available than rat models. For instance, mouse agrin knock-out models were used to prepare co-cultures of chick ciliary ganglion cells and agrin-deficient myotubes [93] as well as agrin-deficient motor neurons and chick myotubes [73]. Another example are HB9:GFP mice, which can be used to prepare co-culture of embryonic GFP-expressing motor neurons and primary mouse myotubes, thus enabling visualization of presynaptic motor neuron terminals apposing nAChR clusters in myotubes [63,149]. The use of the mouse-human co-culture would therefore enable the usage of transgenic mouse models to interrogate the function of various proteins that are important for motor neuron or NMJ function and to uncover molecular mechanisms underlying pathogenesis of neuromuscular diseases. An alternative solution is to express wild type or mutant proteins in co-cultured cells by introducing plasmids with lipofectamine-mediated transfection or electroporation [108]. Another interesting possibility is to use the iPSC technology to generate human myotubes carrying specific mutations [150]. Similarly, fibroblasts and other non-muscle cells, obtained from patients with genetic diseases, can be transformed into myotubes by transfecting them with genes that regulate myogenesis [151]. Suchapproaches may be used to interrogate involvement of specific proteins in pathogenesis of neuromuscular diseases, such as the amyotrophic lateral sclerosis [108,150] and muscular dystrophy [151]

### 2.3. Characterization of the Neural Component of the Co-Cultures of Human Skeletal Muscle Cells and Rat Spinal Cord Explants

Motor neurons that extend their axons from embryonic rat spinal cord explants form highly differentiated, functional and long-lived NMJs. Conversely, dissociated neurons from either chick [152] or mouse [89] spinal cords form neuromuscular contacts that are not only morphologically and functionally immature, but also short-lived [89,90]. Dissociated motor neurons co-cultured with skeletal muscle cells do not survive long enough to allow formation of mature NMJs [89,153]. Under in vivo conditions, segmental and suprasegmental afferents play an important role in survival of motor neurons [154]. However, the loss of such afferent inputs probably does not provide the sole explanation for poor survival of dissociated motor neurons in culture. Indeed, while motor neurons in explants of embryonic rat spinal cord also lack all suprasegmental and the majority of segmental afferent inputs, they are nevertheless capable of forming mature and long-lived NMJs. Major functional differences among various co-culture models can be explained by the presence or absence of glial cells, sensory cells and neural networks.

#### 2.3.1. The Essential Role of Glial Cells and Sensory Neurons

Glial cells, such as Schwann cells, play major roles in differentiation, maturation and long-term survival of motor neurons as well as maintenance of NMJs [155] (reviewed in [156,157,158]), indicating that the presence of glial cells may promote survival and function of motor neurons in culture. This notion is indirectly supported by observation that differentiation of astrocytes, Schwann cells and oligodendrocytes in the rat-human co-culture model progresses in parallel with the differentiation of motor neurons and NMJ synaptogenesis [46]. Furthermore, motor neurons that lack interactions with glial cells are not sufficiently differentiated to establish functional NMJs [155,156]. In co-cultures of *Xenopus* embryonic spinal neurons and skeletal muscle cells, survival of dissociated neurons can be increased by a cocktail of growth factors, but such artificial growth support results in lower synaptogenetic potential, as assessed by reduced nAChR clustering [159]. Synaptogenetic potential can be restored by adding the Schwann cell-conditioned medium containing transforming growth factor-β1 [160], which again underscorses the importance of glial cells.

Aside from glial cells, connections with sensory neurons are also important for the function of rat motor neurons in spinal cord explants. Indeed, the presence of dorsal root ganglia, where perikarya of primary sensory neurons reside, is essential for successful innervation of human myotubes [25]. Importantly, contractions of cultured myotubes emerge only if motor neurons, Schwann cells and sensory neurons are added to cell culture together [45]. Taken together, these findings show that survival and maturation of motor neurons as well as formation of morphologically and functionally well-developed NMJ in vitro are dependent on glial and sensory cells. Thus, absence of these cells is likely a major reason why dissociated motor neurons cannot establish functional NMJs when co-cultured alone with skeletal muscle cells [89]. However, as evident by establishment of functional NMJs between the iPSC-derived human motor neurons and primary human myotubes [50], recent developments in the iPSC technology may have overcome some of the shortcomings associated with using dissociated primary motor neurons. For instance, co-culture of the iPSC-derived motor neurons and myotubes can be used to study neuromuscular disorders, such as myasthenia gravis [50].

#### 2.3.2. Neural Networks and Spontaneous Neural Activity in Spinal Cord Explants

A corollary to the presence of motor neuron-driven contractions of human myotubes in the rat-human co-culture model is that rat motor neurons are spontaneously active. The simplest explanation for this would be the absence of suprasegmental inhibitory inputs to the motor neurons. According to this idea spontaneous activity of motor neurons would be analogous to increased muscle tone and hyperreflexia in patients who are spastic after spinal cord injury or stroke. In these patients, spontaneous firing of action potentials in motor neurons is explained by neuroplasticity and the lack of inhibitory signals from the supraspinal areas of the central nervous system [84,161]. In the homologous rat-rat model strychnine and/or bicuculline, which block the action of inhibitory neurotransmitters glycine and GABA, respectively, convert asynchronous contractions of individual myotubes into episodic, rhythmic and tetanic contractions of bundles of myotubes [68]. This indicates not only that motor neurons receive local inhibitory inputs, but also that embryonic spinal cord explants contain neural networks which display rhythmic activity. These networks, which have been described in detail elsewhere [68,121,162,163,164,165,166], seem to reflect the presence of spinal rhythm generators [167] and are consistent with spontaneous bursts of activity in embryonic spinal cord [168].

### 2.4. Formation of the Basal Lamina in the Synaptic Cleft coincides with the Transformation of Immature Neuromuscular Contact into Differentiated NMJ

Mature NMJ contains basal lamina in the synaptic cleft. As mentioned before, basal lamina provides structural support and binding sites for synaptic molecules, such as AChE and agrin [2,3,29]. During synaptogenesis in co-cultures, synaptic basal lamina starts to form approximately after 10 days (Table 1). At this point first dense patches, which are typical for its structure, appear at the neuromuscular contacts [24]. Appearance of synaptic basal lamina approximately coincides with transformation of immature, bouton-like, neuromuscular contacts into differentiated NMJs, which are characterized by mature nAChR clusters, subsynaptic accumulation of myonuclei and dense patches of AChE [27]. Concurrent formation of basal lamina between the presynaptic and postsynaptic membrane and maturation of NMJ observed in our co-cultures is in agreement with the view that appearance of basal lamina is a key event in NMJ synaptogenesis [169] and with the observation that it determines the location of AChE in the synaptic cleft of NMJ [170]. 

## 3. The Expression of AChE during NMJ Formation in Co-Cultures of Human Skeletal Muscle Cells and Embryonic Rat Spinal Cord Explants.

Understanding formation of the NMJ requires also an answer to the question of the cellular origin of the NMJ components. Some of these components are expressed either exclusively in the skeletal muscle or the motor neuron and their origin is therefore clear. However, some NMJ components, such as AChE and agrin are expressed in both, skeletal muscle and motor neuron. Specific isoforms of AChE and agrin are secreted into the extracellular space and subsequently bound to synaptic basal lamina, which means that they might originate in motor neuron and/or myofiber. 

The question regarding the muscle *vs*. neuronal origin of junctional AChE was investigated by several authors using different experimental approaches and models. Muscle origin of AChE in the NMJ has never been disputed as it is supported by strong evidence [170,172,173]. It is now accepted that great majority of AChE, in the NMJ belongs to A12 AChE, the asymmetric collagen-tailed form, in which three tetramers of tailed (T) variant of AChE catalytic subunit are bound to the triple helical strands of collagen Q (ColQ) structural subunit. This subunit enables AChE binding to the perlecan [174,175], a component of the synaptic basal lamina (reviewed in [3,5]). It is known that both the catalytic [176] and the structural ColQ subunits [177] of asymmetric AChE molecular forms are both expressed in the myonuclei at the NMJ. A minor part of AChE in the NMJ belongs to AChE tetramers linked to plasma membrane of both skeletal muscle and motor nerve terminal with another structural subunit, a small transmembrane protein named proline rich membrane anchor (PRiMA) [172,178,179]. Although minor in comparison to ColQ bound AChE, this portion of tetrameric AChE seems to serve for the fine tuning of the AChE level as its expression is controlled by muscle activity [180,181,182,183]. Interestingly, PRiMA-bound AChE is high in the extrajunctional regions [172,184] opening again a question of AChE function in the areas where other cholinergic components are practically absent.

While skeletal muscle origin of AChE is undisputed, there are several lines of evidence which suggest that motor neurons may also contribute to AChE in NMJ. Expression of AChE in the motor neurons has been demonstrated a long time ago [185]. Consistent with this finding, Anglister [186] demonstrated de novo synthetized AChE in the NMJ basal lamina in the frog experimental model, in which the skeletal muscle fiber part of NMJ was eliminated while the nerve terminal remained intact. Furthermore, AChE in the synaptic cleft is localized closer to the nerve terminal than to the postsynaptic membrane [187]. Besides, ColQ mRNA is also expressed in the spinal cord explants [122,188] and most of the AChE along the nerve terminal is anchored by ColQ, while only a small fraction is anchored by the neuronal membrane anchor PRiMA [172]. Furthermore, tailed (T) catalytic subunits and structural ColQ subunits of AChE are co-expressed in the perikarya of motor neurons [189]. Notably, motor neurons are actually the only producers of the tailed (T) AChE catalytic subunits in the ventral horns of the rat spinal cord [188]. In addition, axons extending from the spinal cord explant and approaching myotubes exhibit strong thiocholine-based [190,191] AChE staining [27,122]. That this AChE activity belongs to the ColQ-bound catalytic subunits was strongly supported by the demonstration of anterograde transport of the asymmetric A12 AChE in the sciatic nerve of rat, chick and rabbit [192,193,194]. All this data clearly supports the view that a part of the AChE synthetized in the perikarya of motor neurons contributes to the basal lamina-bound AChE in NMJ. 

Evidence supporting neuronal origin of AChE in the NMJ was also obtained in co-cultures of embryonic rat spinal cord and human skeletal muscle cells, which provided an alternative approach to tackle the question of the cellular origin of AChE in the NMJ. Unlike some other experimental approaches, in which neuronal or muscular component was removed [170,186] or NMJs were more immature [195], the rat-human co-cultures contain well-differentiated NMJs comprising the presynaptic and postsynaptic components of rat and human origin, respectively [24]. By using species-specific antibodies, we were able to distinguish between muscular (i.e., human) and neuronal (i.e., rat) AChE. As assessed by immunocytochemistry, human-specific anti-AChE antibodies resulted in a particularly strong signal [122], consistent with skeletal muscle being the major source of synaptic AChE [170,172,173]. Immunocytochemical staining of AChE in NMJ was weaker when the rat-specific anti-AChE antibodies were used, but it was nevertheless clearly recognizable [122]. Thus, motor neurons are apparently capable of contributing a fraction of synaptic AChE at least in NMJs formed by rat motor neurons and human myotubes in vitro. 

Expression pattern of AChE and synaptogenetic (z8 and z19) and non-synaptogenetic (z0) agrin isoforms (for nomenclature see subchapter 4 below) in the spinal cord provides additional, albeit indirect, support for the notion that motor neurons contribute to the basal lamina-bound AChE [8]. As assessed by in situ hybridization and PCR, expression of the synaptogenetic agrin-z8 isoform and ColQ, which enables AChE binding to basal lamina of NMJ, increase in parallel after NMJ basal lamina is formed. Based on the assumption that neuronal proteins that are targeted to basal lamina have similar expression patterns and are expressed within a similar time-frame, this result indirectly suggests that neuronal AChE is delivered to NMJ. Consistent with this notion, expression pattern of muscle agrin-z0, which lacks synaptogenetic activity, was completely different from that of agrin-z8. Expression patterns of catalytic AChE subunits and agrin-z19 differed from patterns of ColQ and agrin-z8, suggesting their central nervous system functions unrelated to the NMJ. These results are compatible with the report of Dobbertin et al. [196], who found that distribution and targeting of AChE in mouse striatum critically depends on the binding to PRiMA. Catalytic AChE subunits synthetized in motor neurons have different functions and must therefore be targeted to different places: targeting is obviously determined by its binding to appropriate structural subunit. Similar temporal patterns of expression of ColQ and synaptogenetic variant of agrin in motor neurons supports the possibility that tailed AChE is delivered to the same target, i.e., basal lamina in the synaptic cleft, as agrin [8].

Results supporting the partial neuronal origin of synaptic AChE are in apparent contradiction with those of Camp et al. [197] and Bernard et al. [172]. In these studies, no AChE was found in the NMJs of mice, in which AChE expression had been selectively eliminated in skeletal muscle. This discrepancy might results from the difference in the experimental models. In the experiments by Camp et al. [197] and Bernard et al. [172], basal lamina was formed without muscular AChE, which is a basal lamina component in the wild-type mice. According to the “molecular parking lot” hypothesis [198], AChE occupies distinct location (“parking lot”) in the basal lamina, which is involved in AChE trafficking so that AChE molecules can be removed from their “parking lot” and replaced by new ones. If synaptic basal lamina is formed in the absence of muscular AChE, it is quite possible that its “parking lot” in the basal lamina scaffold is absent or structurally deformed to the point which does not allow AChE of the neuronal origin to bind to this site. 

It is worth mentioning that NMJs in the AChE knockout mice exhibit morphological as well as functional differences in comparison to the wild-type mice [199]. Furthermore, data obtained in knock-out mice models should be treated with some degree of caution because these mice sometimes exhibit characteristics that do not relate to physiology in their wild-type counterparts. A good example of such discrepancy is the AChE knock-out mouse [200,201], which is born alive although wild-type newborns and adults die if AChE is inhibited. According to Bernard et al. [172], the nerve-derived AChE in the NMJ belongs only to the PRiMA-anchored tetramers, which is in disagreement with the previously mentioned anterograde transport of the ColQ-bound asymmetric A12 AChE in the sciatic nerve of rat, chick and rabbit [192,193,194]. These studies did not demonstrate that the ColQ-bound asymmetric A12 AChE accumulates at the synaptic basal lamina. However, while more recent studies do not support the idea that this A12 AChE is delivered to NMJ [172], existence of anterograde transport of AChE in motor neurons still requests the answer to the question where this transportation is destined to. 

In sum, aside from skeletal muscle, AChE in NMJ seems to partially originate from motor neurons. Skeletal muscle is the predominant, but likely not the exclusive, source of AChE in mammalian NMJ. What is the biological meaning of having an additional, neuronal, source of AChE remains an open question. As evident from clinical cases of poisoning with organophosphorous compounds, AChE inhibition in the NMJ is lethal and its absence [202] or absence of ColQ causes myasthenic syndrome [202,203]. Importantly, a recent study showed that exercise training increases synaptic AChE activity. The increase was due to elevated expression of membrane-bound G4 AChE isoform in myofibers [183]. Expression of the basal lamina-bound A12 isoform, which is the most important NMJ isoform [5,71], remained unaltered, indicating that other AChE isoforms dynamically respond to physiological stimuli and actively contribute to NMJ function. By extension, we can speculate that contribution of synaptic (basal lamina-bound) AChE from both myofibers and motor neurons might also have a role in fine-tuning the neuromuscular transmission. Alternatively, dual origin of AChE might represent a safety factor against malfunctions due to impaired AChE turnover in either skeletal muscle or motor neuron. 

## 4. The Role of Neural Agrin in the Formation of the NMJ in the in Vitro Innervated Human Skeletal Muscle Cells

Homologous and heterologous co-culture models, which used various combinations of neural and skeletal muscle cells, had an important role in establishing that nerve-derived factors promote aggregation of nAChRs and differentiation of postsynaptic membrane [23,33,92,204,205,206,207,208]. Investigations of NMJ synaptogenesis culminated in discovery of agrin and its biological functions [4,209,210,211,212,213,214,215,216]. The neural isoform of agrin, which plays a major role in formation and maintenance of NMJ, contains an 8, 11, or 19 amino acid insert at the z-site (called the B-site in avian species) [217,218,219,220,221]. The insert at the z-site enables neural agrin to bind to Lrp4 and activate MuSK, which in turn promotes clustering of nAChRs and organization of other NMJ components [1,43,44]. 

The co-culture approach proved to be very useful for investigation of agrin action in vitro. Indeed, very early on in the agrin research several co-culture models using chick, rat and frog spinal cords and skeletal muscle cells were used to assess its nAChR aggregating activity [222,223,224,225]. For instance, the heterologous rat-chick model was used to investigate the role of muscle agrin [123], which lacks an amino acid insert at the z site (agrin-z0) and, compared with neural agrin, displays nAChR aggregating activity only at high concentrations [73,219,221,222]. Using this heterologous model and species-specific antibodies, muscle agrin was shown to be present in the NMJ, which indicated that muscle agrin may have a role in NMJ independent of nAChR aggregation [123]. Consistent with this notion, chick muscle agrin (agrin-B0) was shown to promote presynaptic differentiation of chick ciliary ganglion cells co-cultured with COS cells [226]. In the same study, the homologous chick model, a co-culture of chick ciliary gangion and skeletal muscle cells, was used to demonstrate that anti-agrin antibody reduces presynaptic as well as postsynaptic differentiation of NMJs [226]. 

In a similar approach, co-cultures of *Xenopus* embryonic neurons and skeletal muscle cells were used to assess the role of α-dystroglycan, an alternative agrin receptor [227,228,229,230,231,232], in NMJ formation [233]. Later research showed that presynaptic differentiation of dissociated chick ciliary ganglion cells was not impaired when they were co-cultured with mouse myotubes lacking agrin [93], indicating that muscle-derived agrin may be dispensable for NMJ assembly. Consistent with this idea, neural agrin is required to prevent activity-dependent dispersal of nAChR clusters in co-cultures of mouse motor neurons and chick skeletal muscle cells, while muscle agrin does not oppose declustering of nAChRs [73]. These studies in various animal co-culture models provided important insights into agrin action; however, they did not examine the effect of agrin on NMJ synaptogenesis in human skeletal muscle cells. 

In mouse embryos NMJ develops in two stages as regards effects of agrin [234]. In the first stage nAChRs clusters form independently of agrin and innervation. In the second stage, neural agrin promotes formation of new nAChR clusters or stabilization of existing nAChR clusters [234]. The rat-human co-cultures were used to assess the role of agrin in the establishment of functional innervation in human skeletal muscle cells. As mentioned above, this model is particularly suited for assessment of functional innervation since all observable contractions of myotubes are the result of innervation and formation of functional NMJs. Furthermore, establishment of functional innervation can be easily quantified by counting the number of the contraction-positive explants and the contracting units (for definitions see Table 1). Treatment of co-cultures with anti-agrin antibody, Agr 33, which blocks the action of neural agrin, reduced the number of nAChR clusters by 80% and their long axes by 50%. Nevertheless, contractions of myotubes were present at 7–10 days of co-culturing. While this result seems to indicate that blockage of agrin action does not prevent establishment of functional innervation, Agr 33 antibody suppressed the increase in the number of contracting units [42]. In contrast, under control conditions, the number of contracting units steadily increases to reach a plateau up to approximately 17th day of co-culture (Table 1). 

Based on these results, establishment of functional innervation in vitro can be divided into the agrin-insensitive and the agrin-sensitive stage, consistent with findings in animal models [234,235]. In the early, agrin-insensitive, stage a small fraction of NMJs become functional (i.e., contraction competent) even if the agrin action is suppressed. Obviously, at these nerve-muscle contacts concentration of nAChR is already sufficient for eliciting end-plate potentials, while the number of Na^+^ voltage-gated channels is sufficient for this endplate potential to subsequently reach the threshold and trigger action potential. However, the majority of NMJs requires agrin to become functional during the later, agrin-sensitive, stage. The two-stage development of functional innervation with regard to agrin effects explains why, in the presence of Agr33 antibody, the number of contracting units did not increase after day 10, although contractions were observed in 7–10 day old co-cultures. This was the first characterization of the contribution of agrin to NMJ development in human skeletal muscle cells [42]. This observation suggested that neural agrin is sufficient as an inducer of NMJ formation at the contact with the skeletal muscle membrane but is not absolutely necessary for the establishment of early primitive neuromuscular contacts between rat motor neurons and human myotubes. This finding is in line with the current model of agrin action, in which MuSK, but not agrin, is required for initial nAChR clustering, while agrin primarily stabilizes existing nAChR clusters and prevents their activity-induced disaggregation [1,6,7,73,235,236]. 

## 5. Possible Alternative Roles of AChE and Neural Agrin in Human Skeletal Muscle

From the standpoints of their classical roles, AChE and agrin have very little in common. One is a cholinergic component and the other a synaptogenic inducer. Traditional view holds that components of the NMJ belong to the two functional groups. The first encompasses proteins that play a role in cholinergic signaling. Aside from AChE, representatives of this group include choline acetyl transferase, which synthesizes ACh, and nAChR, which transduces ACh signal (reviewed in [237]). The second group encompasses proteins that underlie synaptogenesis and maintenance of NMJ. Aside from neural agrin, major representatives of this group are agrin receptor Lrp4 and MuSK, which triggers downstream signaling cascade leading to NMJ differentiation (reviewed in [1,38]). While useful, this simple functional division does not fully reflect molecular characteristics of AChE and agrin. 

Members of the same group differ in many respects. For instance, while AChE and nAChR both bind ACh, they have dissimilar expression patterns, protein structure and even the ACh binding site. Also, AChE and agrin share several features despite belonging to different functional groups. First of all, they are both polymorphic. Indeed, while AChE and agrin exist in several different variants, they are both a product of a single gene. Alternative splicing is therefore a major mechanism underlying functional diversity of AChE and agrin variants. Another similarity between AChE and agrin is that, unlike most other NMJ components, they are expressed in motor neuron as well as in skeletal muscle. Furthermore, AChE [238] and agrin [7] are expressed in various other types of cells, where they play physiological roles that are not related to cholinergic neurotransmission or synaptogenesis and maintenance of NMJ. Finally, both AChE and agrin apparently play non-canonical, alternative, roles during myogenesis and skeletal muscle regeneration, such as apoptosis [239] and differentiation of primary human skeletal muscle cells [8,82,240], respectively. 

### 5.1. AChE and Apoptosis of Primary Human Myoblasts

As well as for the generation of new cells, normal tissue turnover requires apoptosis to remove cells that are senescent or do not function properly [241]. On the other hand, excessive apoptosis of myogenic cells reduces regeneration capacity, thereby impairing skeletal muscle function [242,243]. Notably, AChE was shown to be involved in regulation of apoptosis of different types of cells, including hematopoietic cells [244], cultured smooth muscle cells, fibroblasts, endothelial cells and various cancer cells [245]. 

AChE is expressed not only in innervated muscle fibers but also in proliferating myoblasts [27], although these cells lack most other components of the cholinergic system. We therefore examined whether AChE might have a similar role during the early stages of skeletal muscle regeneration in vitro. We made two major observations. First, siRNA-mediated silencing of AChE in primary human myoblasts reduced staurosporine-induced activity of the initiator caspase 9 and the executioner caspase 3/7 [239]. Consistent with reduced caspase activity, AChE silencing reduced the fraction of apoptotic myoblasts as assessed with annexin V/propidium-iodide and 3,3-dihexyloxacarbocyanine iodide (DiOC_6_(3)) staining. Second, staurosporine differentially affected AChE variants T, H and R; it induced an 8-fold increase in AChE-R mRNA expression in myoblasts and a statistically non-significant 2-fold increase in AChE-T mRNA, but it did not alter expression of AChE-H mRNA. These data suggest that AChE-R variant might play a key role in modulation of myoblast apoptosis. However, on the other hand, in cultured myoblasts AChE-T is the most abundant AChE isoform and represents a major fraction of the total AChE activity [132]. A small increase of its expression may therefore have a greater impact on myoblast apoptosis than a marked increase in expression of AChE-R. 

Although direct stimulation of apoptosis by AChE was noted in some cases [246], increased AChE expression per se is not necessarily sufficient to directly trigger apoptosis. Thus, AChE promotes apoptosis primarily in conjunction with other apoptotic stimuli [11], such as staurosporine [239]. The mechanism by which AChE modulates apoptosis in myoblasts and other cells is unclear. One possibility is that AChE promotes apoptosis indirectly by hydrolyzing ACh, which would tend to decrease pro-survival cholinergic signalling via nAChR and/or muscarinic AChR [247]. For instance, AChE inhibitors, such as tacrine and physostigmine, decreased apoptosis in fibroblasts [245], thereby suggesting that the catalytic activity of AChE might be linked to regulation of apoptosis. However, AChE inhibitors failed to protect against apoptosis of Jurkat cells [248]. Indirect, ACh-dependent, mechanism might therefore be important for pro-apoptotic effects of AChE under special circumstances, such as in ACh-secreting cells or in the presence of exogenous ACh, but they likely do not represent the most important mechanism by which AChE modulates apoptosis. Consistent with this view, nAChR agonist carbachol was unable to prevent apoptosis in glioblastoma cells [246]. Furthermore, C-terminal fragment of AChE [249] as well as AChE that possesses no cholinesterase activity both promote apoptosis [250], underscoring the separation of cholinesterase and proapoptotic functions of AChE. Pharmacological inhibitors of AChE, when effective, may therefore reduce apoptosis by inducing structural changes, which subsequently suppress its pro-apoptotic activity, and not by reducing cholinesterase activity per se [246].

Importantly, AChE-T variant was recently shown to possess DNase activity [250]. AChE inhibitors or mutation of the catalytic triade residues (S234A, E365A and H478A) abolish cholinesterase activity but not DNase activity [250]. This discovery suggests a mechanism whereby AChE translocates into the nuclei of apoptotic cells and directly contributes to degradation of DNA [250]. Furthermore, since AChE-T is capable of translocation into the nucleus [250,251], while AChE-R apparently is not [251], we may speculate that different AChE isoforms or differences in their localization insubcellular compartments might have complementary modulatory functions in apoptotic cells. For instance, the nuclear fraction of AChE-T may contribute to DNA degradation and laddering, while its cytoplasmic fraction or one of the other AChE isoforms may promote apoptosis by interacting with Apaf-1, cytochrome c or other regulators of apoptosis [252,253]. Taken together, although the underlying mechanisms should be characterized in more detail, current evidence strongly supports involvement of AChE in modulation of apoptosis [10,11]. Indeed, AChE is even regarded as a marker of apoptosis [10,248].

In most cells, which play no role in cholinergic transmission, AChE is expressed and active only once these cells enter apoptosis [245]. In contrast, AChE is prominently expressed and active in proliferating myogenic precursors, such as myoblasts [132,239], as well as in mature myofibers. Involvement of AChE in termination of neuromuscular transmission as well as apoptosis of myogenic precursors raises the question how these two divergent biological functions are reconciled. Balance between AChE functions might be maintained by their temporal and spatial separation. For instance, during the early stages of myogenesis or muscle regeneration, when cholinergic components are not developed, AChE might be important for regulation of myoblast survival and apoptosis. To promote apoptosis, AChE should be localized in cytoplasm or nucleus of myoblasts, which is indeed the case [27]. In apoptotic myoblasts, AChE might be redirected from the secretory route, which delivers AChE to the extracellular space, to nucleus, cytoplasm or other subcellular compartments, where it can exert its pro-apoptotic functions. Conversely, in later stages of skeletal muscle regeneration, during synaptogenesis and in mature myofibers, redistribution of AChE to the secretory route would tend to increase its delivery to the NMJ, while suppressing its pro-apoptotic function. Thus, regulation of subcellular distribution of AChE in different developmental stages may provide a mechanism by which AChE performs two divergent functions in skeletal muscle.

### 5.2. Neural Agrin and Differentiation of Cultured Human Skeletal Muscle Cells

Biological significance of alternative, non-cholinergic, roles of AChE has been subjected to different interpretations, although these roles were demonstrated in various experimental systems. While this discussion regarding alternative roles of AChE continues, multitasking proteins have emerged as a common theme in biology of enzymes and various other proteins [254,255]. One example are the metabolic enzymes performing functions that are unrelated to their catalytic activities. For instance, lactate dehydrogenase, which interconverts pyruvate and lactate, is also a structural component of duck lens [256], while aconitase, which interconverts citrate and isocitrate in the Krebs cycle, can act as an RNA binding protein that is involved in iron homeostasis [257]. Another example of a multitasking protein is agrin, which was discovered as an α-motor neuron-derived heparan-sulfate proteoglycan that plays a major role in formation and maintenance of NMJ [4,7]. Later research showed that agrin plays additional roles in immune system [7], cancer [258] and, most recently, heart regeneration [259]. Non-neural isoforms of agrin and/or alternative agrin receptors, such as α-dystroglycan or integrins [227,228,229,230,231,232,260], are likely responsible for many of these additional roles [7]. Nevertheless, neural agrin and the canonical Lrp4/MuSK pathway promote proliferation of liver cancer cells [258], indicating neural agrin is also involved in functions that are not related to NMJ. 

In skeletal muscle, the role of agrin is also not limited exclusively to its functions in NMJ. Indeed, we showed that agrin promotes different aspects of myotube maturation. First, neural agrin increases the fraction of human myotubes that raise intracellular Ca^2+^ concentration in response to depolarizing solution (60 mM KCl) or caffeine (40 mM). Furthermore, agrin increases the density of L-type Ca^2+^ currents, indicating that it promotes the development of excitation-contraction coupling [82]. In contrast to human myotubes, in murine myotubes neural agrin does not alter excitation-contraction coupling [82], again underscoring functional differences between human and rodent skeletal muscle cells. Second, neural agrin makes the resting membrane potential of human myotubes more negative. In addition, strophantidin, an inhibitor of Na^+^/K^+^-ATPase, depolarizes agrin-treated myotubes more than control myotubes [240]. These effects are paralleled by increased expression of α1- as well as α2-subunit of Na^+^/K^+^-ATPase [240]. Taken together, these data indicate that in cultured myotubes, neural agrin increases both expression and activity of Na^+^/K^+^-ATPase, which plays a major role in skeletal muscle physiology [261,262]. Third, we also showed that neural agrin increases the expression of slow and fast MyHCsin cultured myotubes [8] and increases secretion of interleukin-6 in human myoblasts as well as in co-cultures with embryonic rat spinal cord [9]. Collectively these data indicate that neural agrin, like AChE, has multiple alternative modulatory roles in addition to its canonical role in NMJ. 

## 6. Conclusions and Perspectives

In this review we demonstrate that the human-rat co-culture model has served as a useful experimental approach for the investigation of NMJ synaptogenesis and the function of innervated human skeletal muscle cells. In addition to basic neurobiology, this model enabled investigation of molecular mechanisms underlying pathogenesis of various neuromuscular diseases using cells obtained from patients. Although these approaches have been validated a long time ago, they are still relevant today. Indeed, the advances in culturing of skeletal muscle cells, such as 3D scaffolds, and in molecular biology, especially the arrival of new powerful techniques, such as RNA interference, CRISPR and the iPSC technology open new opportunities to address unresolved issues regarding AChE and agrin function in human skeletal muscle as well as NMJ neurobiology in general. Conventional models of in vitro innervation of human skeletal muscle cells combined with these new methodologies represent a powerful set of complementary experimental tools to uncover new molecular mechanisms underlying NMJ development and function, which may lead to discovery of new pharmacological targets and development of novel therapeutic options for neuromuscular diseases.

## Figures and Tables

**Table 1:** Developmental characteristics of functional innervation in co-cultures of rat spinal cord and human skeletal muscle cells. A contracting unit is a distinct group of cultured myotubes (myofibers) that contract simultaneously at a frequency that is different from the frequencies of other contracting units. Contraction-positive explant is an explant that established functional neuromuscular contacts (i.e., has at least one contracting unit). Presented data are based on: [27,42,46,122,171]. Staging of co-cultures is based on the appearance of basal lamina [24,171].

Co-Culture Stage	Basal Lamina	Formation of Functional Neuromuscular Junctions	Acetylcholinesterase and nAChR
Stage I (Day 1–9)	Not formed	First neurite-myotube contact (Day 3)	Diffuse AChE staining in myotubes and neurites extending from the spinal cord explant
		First α-bungarotoxin-sensitive contractions occur (Day 7)	AChE expressed in all myonuclei and present along the whole myotube length
		No visible cross-striations in myotubes	Immature nAChR clusters at neuromuscular contacts
Stage II (Day 10–21)	Formed	Number of contraction-positive explants attains plateau (Day 10)	AChE expressed predominantely at junctional myonuclei
		Number of contracting units attains plateau (Day 17)	Discrete patches of AChE originating in motor neurons and myotubes
		Cross-striations visible in contracting myotubes	Mature nAChR cluster at neuromuscular contacts AChE and nAChR co-localize in discrete patches
